# Effects of early and late cheiloplasty on anterior part of maxillary dental arch development in infants with unilateral complete cleft lip and palate

**DOI:** 10.7717/peerj.1620

**Published:** 2016-02-15

**Authors:** Silvia Valentová-Strenáčiková, Radovan Malina

**Affiliations:** 1 Clinic of Plastic, Reconstructive and Aesthetic Surgery, F. D. Roosevelt Hospital, Banská Bystrica, Slovakia; 2 Department of Biology and Ecology, Matej Bel University, Banská Bystrica, Slovakia

**Keywords:** Complete unilateral cleft lip and palate, Early and late lip reconstruction, Dentoalveolar arch development, Cheiloplasty

## Abstract

**Objectives.** The objective of this study is to compare the impact of early and late reconstruction of complete unilateral cleft lip and palate on the growth and development of the front of the dentoalveolar arch. **Methods.** This study was carried out in the years 2012–2015 at the Clinic of Plastic, Reconstructive and Aesthetic Surgery in Banska Bystrica. Infants with unilateral complete cleft lip and palate were divided into 2 groups according to the timing of lip reconstruction. Group A consisted of infants with early lip reconstruction–realised in the first 14 days of life. Group B consisted of infants with later lip reconstruction–realised in the third month of age. Maxillary dental casts were obtained for each child in four periods–in the first 14 days of life, in the third month, in the sixth month and in the age of one year. These were followed by the identification, measurement and evaluation of anthropometric parameters. **Results.** Significant differences were occurred after the reconstruction of the lips in linear and angle measurements between infants in the A and B groups. **Conclusion.** The early surgical reconstruction of the lips in the first 14 days of life has a positive effect on the growth and development of the anterior segment of the dentoalveolar arch. Early lip reconstruction forms a continuous pressure on the frontal segment, resulting in the earlier remedy of anatomical properties and creates appropriate conditions for the best development of this area.

## Introduction

Cleft malformations belong to the most common facial congenital defects. The patients are affected by cleft both aesthetically and functionally. Final result of cleft treatment depends on the appropriate choice and timing of the surgical and conservative methods. Cleft treatment is very difficult and lasts from the birth to the adulthood. We should focus on the close correlation of morphological and functional aspects in the treatment of clefts.

There are many different opinions on the lip reconstruction timing. Nowadays the lip repair is possible already in the first week after birth (Desai, 1997; Galinier et al., 2008; Le Pendeven, Martinot-Duquennoy & Pellerin, 2009; Harris et al., 2010). Generally, the surgical reconstruction of cleft lip and palate is performed from the first hour of life to adulthood (Mazaheri et al., 1971; Millard, 1976; Bromley, Rothaus & Goulian, 1983). The early surgical lip reconstruction does not result in the increasing of perioperative mortality or neonatal morbidity and the result is comparable with later reconstruction (Burt & Byrd, 2000). Calteux et al. (2013) also stated very low risk of anaesthetic and surgical interventions limited to the lip before the age of 28 days and a very low rate of complications.

Furthermore, in the first days after birth persists the fetal scarless wound healing. Fetal wound healing is fundamentally different than postnatal healing. Healing of primarily closed, linear wounds occurs rapidly and without scarring. Acute inflammation is not involved, fibroblast recruitment and proliferation is minimal and collagen deposition is highly organized so that scarring is minimal or nonexistent (Mast et al., 1992).

Several studies have shown that fetal skin fibroblasts display major differences at migration, contraction, and secretion from adult fibroblasts, when cultured under identical conditions in vitro (Cullen et al., 1997; Ellis & Schor, 1998). In adults the healing process results in replacement of normal skin structures with scar tissue, but shortly after birth a reduced scar formation is observed (Krejci et al., 2014). This short afterbirth period may be used for lip reconstruction with minimal (or no) scar formation.

Since 1995, we have started with early lip reconstruction at our clinic. Different healing process together with a positive psychological effect, better sucking and better speech development were the main reasons for this decision. Since then, we have operated infants early but also in the age of 3 months (late reconstruction). Late reconstruction was used in patients where the early reconstruction could not be realised because of some actual medical reasons (mostly respiratory infection). The years of experience with both ways of reconstruction (early and late) are the main reason why we decided to deal with the issue of early cleft lip reconstruction in more detail.

The aim of this study was to compare and quantitatively analyze the development of the anterior part of the maxillary dental arch after the early and late reconstruction of the complete unilateral cleft lip and palate.

## Materials and methods

The presented study was realised in the years 2012–2015 at the Clinic of Plastic, Reconstructive and Aesthetic Surgery in Banská Bystrica.

The sample consisted of maxillary dental casts of 35 infants with complete unilateral cleft lip and palate. All infants were born after the 37th week of pregnancy. Maxillary dental casts were taken of these 35 infants in four periods–at the age of 14 days, 3, 6 and 12 months. These casts were taken as a part of medical treatment protocol when necessary. The first casts were taken either during the lip reconstruction (group A) or during the first visit at our clinic (group B). All casts were divided into 2 groups according to the timing of the lip reconstruction.

Group A consisted of casts of 25 infants with early lip reconstruction (reconstruction was performed in the first 14 days of their life). This group consisted of 13 boys and 12 girls. Left-sided cleft was presented in 16 infants and the right-sided in 9 infants. Only the children in excellent health condition without an associated inborn defect were included in this group.

Group B consisted of casts of 10 infants with later lip reconstruction (reconstruction was performed at the age of 3 months). The group consisted of 5 boys and 5 girls. Left-sided cleft occurred in 4 infants and the right-sided in 6 infants. Early lip reconstruction could not be performed in this group due to acute respiratory infections. The pre-surgical maxillary orthodontic treatment was used to reduce the alveolar gap before cheiloplasty in this group.

The complete cleft was surgically solved in two stages. The first stage was the reconstruction of lip and nose using the Millard’s technique with the reconstruction of the nasal wing. The second stage included the palate reconstruction using four flap palatoplasty technique of Wardil-Kilner and was performed at the age of 6–8 months. All reconstructions were performed by the same surgeon.

**Figure 1:**
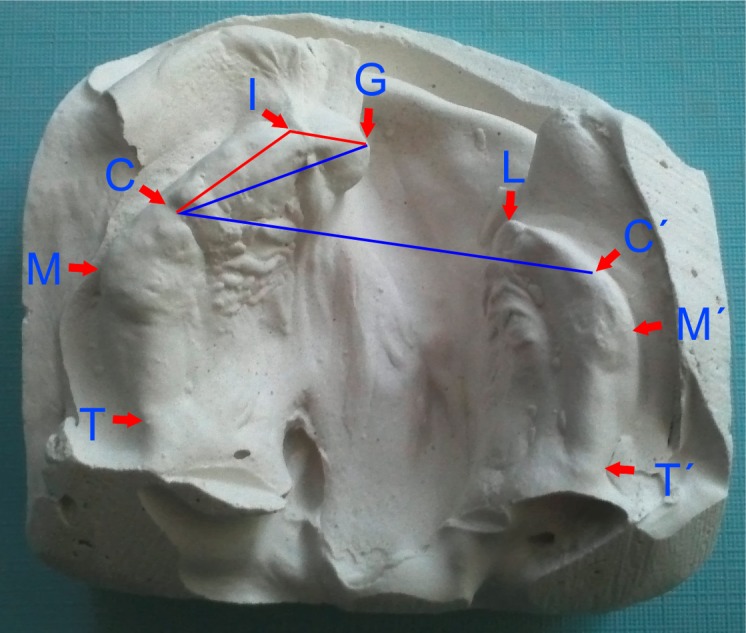
Identification of individual landmarks used for linear and angular (GIC-red, GC-CC′-blue) measurements.

The anthropometric points (Fig. 1) were identified on each dental cast. To analyze the development of the posterior part of the maxillary arch we used standard anthropometric parameters according to Mazaheri et al. (1971). We measured the following linear and angular distances:

G-L alveolar cleft width;
I-G anterior portion of nonclefted segment;
I-C anterior ridge length of nonclefted segment;
GC-CC anterior basal angle;
GIC anterior arch curvature angle on nonclefted segment.

Linear and angular measurements were realised with a digital slide caliper with the accuracy of 0.1 mm, and a goniometer with the accuracy of 0.5°. Each dimension was measured by three examiners and the average value was determined to minimize possible errors. The sets of measurements of one examiner were not available to the others.

The PASW Statistics 18 (SPSS Inc., Chicago, IL, USA) was used for statistical analyses. First, the normality of data was verified by Shapiro-Wilk W test. The data with the normal distribution were analysed by ANOVA followed by Tukey test, remaining data were analysed by the nonparametric Wilcoxon test. The significance level was established at α = 0.05.

## Results

**Figure 2:**
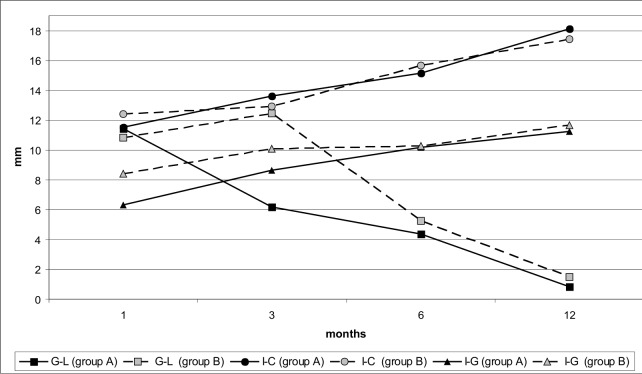
Changes in linear lengths G-L, I-C and I-G of maxillary dental arch during the observed period in infants with the early (group A) and late (group B) lip reparation.

The alveolar cleft width (G-L) was continuously decreasing during the observed period in the group A. The average distance of G-L was 11.42 mm before the lip reconstruction (Table 1). The steepest decline (nearly 50%) was observed in the first three months. Then the G-L distance decreased continuously up to 12 months of age (Fig. 2).

**Table 1:** Linear and angular measurements of maxillary dental arch during observed period in infants with the early (A) and late (B) cheiloplasty.

	In the first 14 days		3 months		6 months		1 year	
	A	B	A	B	A	B	A	B
G-L (mm)	11,42 ± 2,18	10,81 ± 1,81	6,18 ± 1,16**	12,46 ± 1,72**	4,36 ± 1,04*	5,24 ± 1,92*	0,82 ± 0,70**	1,49 ± 0,28**
I-G (mm)	6,31 ± 1,57**	8,43 ± 0,69**	8,64 ± 0,56**	10,07 ± 0,55**	10,19 ± 0,87	10,25 ± 0,75	11,22 ± 0,43	11,64 ± 0,92
I-C (mm)	11,52 ± 1,15*	12,42 ± 0,49*	13,59 ± 0,63*	12,9 ± 0,38*	15,15 ± 0,64*	15,67 ± 0,92*	18,10 ± 0,47**	17,44 ± 0,91**
GC-CC′ (°)	27,42 ± 1,02	28,04 ± 0,90	20,53 ± 0,65**	29,94 ± 0,55**	16,25 ± 0,37**	17,93 ± 0,65**	10,91 ± 0,50	11,37 ± 0,87
GIC (°)	157,33 ± 0,85*	156,65 ± 1,00*	151,25 ± 0,87**	168,33 ± 1,01**	140,44 ± 0,97**	148,64 ± 0,77**	138,33 ± 0,62**	140,91 ± 0,66**

**Notes:**

* The difference between the early (A) and late (B) cheiloplasty is significant (p < 0.05).

** The difference between the early (A) and late (B) cheiloplasty is highly significant (p < 0.001).

In the group B, the alveolar cleft width (G-L) was slightly increasing up to 3 months of age. Then it was significantly decreasing from 3 to 6 months as well as from 6 to 12 months (Fig. 2).

There was no significant difference in the cleft width (G-L) between the groups A and B at the age of 14 days. The G-L width was increasing in the group B during the first 3 months of life. The differences between the groups A and B were continuously reducing after the lip reconstruction in the group B, but remained significant until the end of the observed period (Table 1).

The anterior portion of the nonclefted segment (I-G) was continuously growing throughout the observed period in the group A.

In the group B, there was a sharp increase in the I-G length during the first 3 months of life. Subsequently, the growth was slowing down and until the 6th month there was only a minimal increase. After the 6th month of life the growth was accelerating again (Fig. 2).

Highly significant differences (p < 0.001) were observed between the groups A and B in this length (I-G) during the first three months of life. Until the age of 6 months the differences diminished and subsequently no significant differences were noticed (Table 1).

The anterior ridge length of the nonclefted segment (I-C) in the group A was continuously increasing throughout the reported period (Fig. 2).

In the group B, there was only a negligible increase in the I-C length during the first three months of life. The lip reconstruction in the 3rd month of life was followed by a rapid growth (Fig. 2).

The distance of I-C showed significant differences (p < 0.05) between the A and B group in all reported periods. At the age of 1 year the length of I-C was already significantly longer (p < 0.001) in the group A (Table 1).

**Figure 3:**
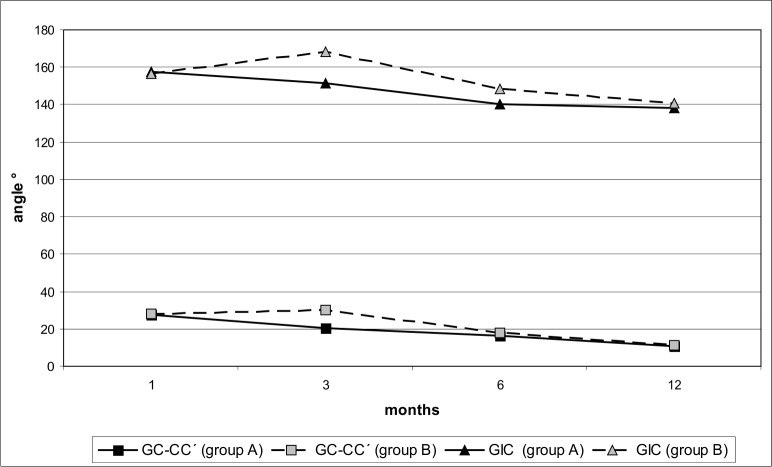
Changes in the size of angles GC-CC′ and GIC of maxillary dental arch during the observed period in infants with the early (group A) and late (group B) lip reparation.

The anterior basal angle (GC-CC′) was decreasing steadily throughout the observed period in the group A. In the group B, the gradual decreasing was observed only after the lip reconstruction (Fig. 3).

Highly significant differences (p < 0.001) were noticed in the size of this angle (GC-CC′) between the groups A and B at the age of 3 and 6 months. In other periods there were no significant differences (Table 1).

The anterior arch curvature angle on nonclefted segment (GIC) in the group A was continuously decreasing throughout the observed period. The most significant decrease was recorded during the first six months. Then was the decreasing reduced (Fig. 3).

In the group B the angle of GIC was significantly increasing during the first three months of life. After the lip reconstruction the angle of GIC was sharply decreasing (Fig. 3).

The measured values of the GIC angle showed statistically significant differences in all reported periods (Table 1).

## Discussion

This study of a group of 35 infants with the complete unilateral cleft lip and palate compared the development of the anterior segment of the dentoalveolar arch of infants with early and late correction of the cleft. Early operation has a significant occlusive effect on alveolar arch (Akin et al., 1991). It has been also reported that primary lip suture in newborns has good aesthetic results concerning lip scars and the appearance of the nose (McHeik et al., 2006; Borský et al., 2007). Further benefits of early neonatal lip repair are very good wound healing and feeding facilitation (Cohen, Marschall & Schafer, 1992).

The evidence of a highly significant difference of the alveolar cleft width (G-L) in the compared groups is considered as the most important finding of this study. The width of the G-L was continuously decreasing from the very early surgical intervention in the group A, while in group the B the G-L width was continuously increasing in the first three months of life. The G-L width in the group A was significantly smaller in the 3rd month than in the group B. The significant difference between the groups A and B maintains until the last measurement at the age of 1 year.

Based on the results we can say that the reduction of the alveolar cleft width (G-L) occurs right after lip reconstruction in both groups. The reconstructions of lip muscular system (orbicularis oris) cause the molding effect on nonclefted segment and enhance the mutual position of the alveolar segments. The same effect was recorded by Huang et al. (2002) and Christie et al. (1991). Also Eichhorn et al. (2011) stated the decreasing of alveolar cleft width shortly after lip closure in 44 clefts.

The anterior ridge length of nonclefted segment (I-C) of infants from the group A was gradually rising throughout the observed period. In the group B the growth of this segment was stagnating during the first three months and began to rise only after the lip reconstruction in the 3rd month. Consequently, this segment was continuously increasing. Such growth was mentioned also by Huang et al. (2002).

A similar effect was also reflected on the anterior portion of the nonclefted segment (I-G). The size of this segment in the group A was continuously expanding throughout the whole observed period. In the group B the growth’s slowing down occurred in this segment between the 3rd–6th months. Then, the growth continued until the end of the observed period. Significant differences between the groups A and B were recorded in the size of the segment I-G at the age of 14 days and 3 months. The differences were negligible in the next measurements. Similar trends of the growth in this segment were noticed by Huang et al. (2002).

The forming of the frontal part of the dentoalveolar arch plays an important role in the first year of life of infants with the complete unilateral cleft lip and palate. The correct position of maxillary arch contributes to a better sucking, feeding and proper development of speech. Therefore, our attention was focused, in addition to the linear measurements, on the evaluation of the angular parameters GIC and GC-CC′. In both cases we observed a gradual reduction of these angles after the surgical reconstruction. Both angles were significantly smaller in the group A than in the group B in the 3rd month of life. This significant difference maintained in the angle of GIC until the end of the reported period. The angle GC-CC′ showed no significant difference in the 1st year of life. A similar phenomenon was observed by Huang et al. (2002), who stated that the angular measurements (GC-CC′, GIC) indicated palatal displacement of the frontal part of the nonclefted segment and the continuous reduction of both angles. The reduction of angles is caused by the molding effect of the lip reconstruction which contributes to the formation and symmetry of the dentoalveolar arch (Eichhorn et al., 2011; Adali et al., 2012).

The reduction of all these values is observed in the group with the early surgical lip reconstruction. The most significant reduction is observed right after the lip reconstruction until the third month. In the group of patients with the late lip reconstruction these parameters are increasing until the 3rd month and only after the reconstruction they are decreasing until the age of 1 year. The results presented by Kramer, Hoeksma & Prahl-Andersen (1994) showed that lip closure performed at 3 months of age has a strong effect in the anterior alveolar region during the next 3 months after the surgery.

There are many opinions of Presurgical Orthodontic Treatment (POT) in the period before the surgical lip reconstruction. Based on the results of many studies and scientific evidence it can be concluded that POT had not a lasting positive impact on the growth of the dentoalveolar arch (Suri & Tompson, 2004; Hsieh, Liao & Shetty, 2012). The POT using in cleft malformations treatment remains largely unsolved problem. In group A we do not use the POT therapy in either case. Its function is overtaken by repaired orbicularis oris muscle. The pressure of this muscle could ensure the proper formation of the dentoalveolar arch and the closure of cleft.

## Conclusion

The results of this study confirm that the early surgical lip reconstruction performed in the first 14 days after birth has a positive effect on the growth and development of the anterior segment of the dentoalveolar arch. The early lip reconstruction forms a continuous pressure on the frontal segment which leads to a faster adjustment of anatomical properties. This pressure creates the appropriate conditions for the best development of this functionally and aesthetically very important area. We have not found out any negative effect on the growth and development of anterior alveolar segment during the first year of life. The early surgical lip reconstruction has provided the optimal position of the alveolar arch segments and there is no need for the presurgical orthodontic treatment. The aesthetics of the middle third of the face, eating, swallowing and also the development of language with the proper phonation were significantly improved in the infants. From this point of view we consider the early lip reconstruction to be better than the later surgery.

The evaluation of the effectiveness of the treatment process is one of the goals of our long-term monitoring program. Patients with the early surgical lip reparation will be monitored and evaluated from birth up to adulthood.

## Supplemental Information

10.7717/peerj.1620/supp-1 Supplemental Information 1 Raw data of linear measurement.

10.7717/peerj.1620/supp-2 Supplemental Information 2 Raw data of angular measurement.

## Additional Information and Declarations

### Data Deposition

The following information was supplied regarding data availability: The raw data is supplied as Supplemental Dataset files.
